# Supplementary figures and images for: Evolutionary history of the vertebrate Piwi gene family
Source: PeerJ. 2021 Nov 5;9:e12451. doi: 10.7717/peerj.12451 (PMC8574217; doi:10.7717/peerj.12451)

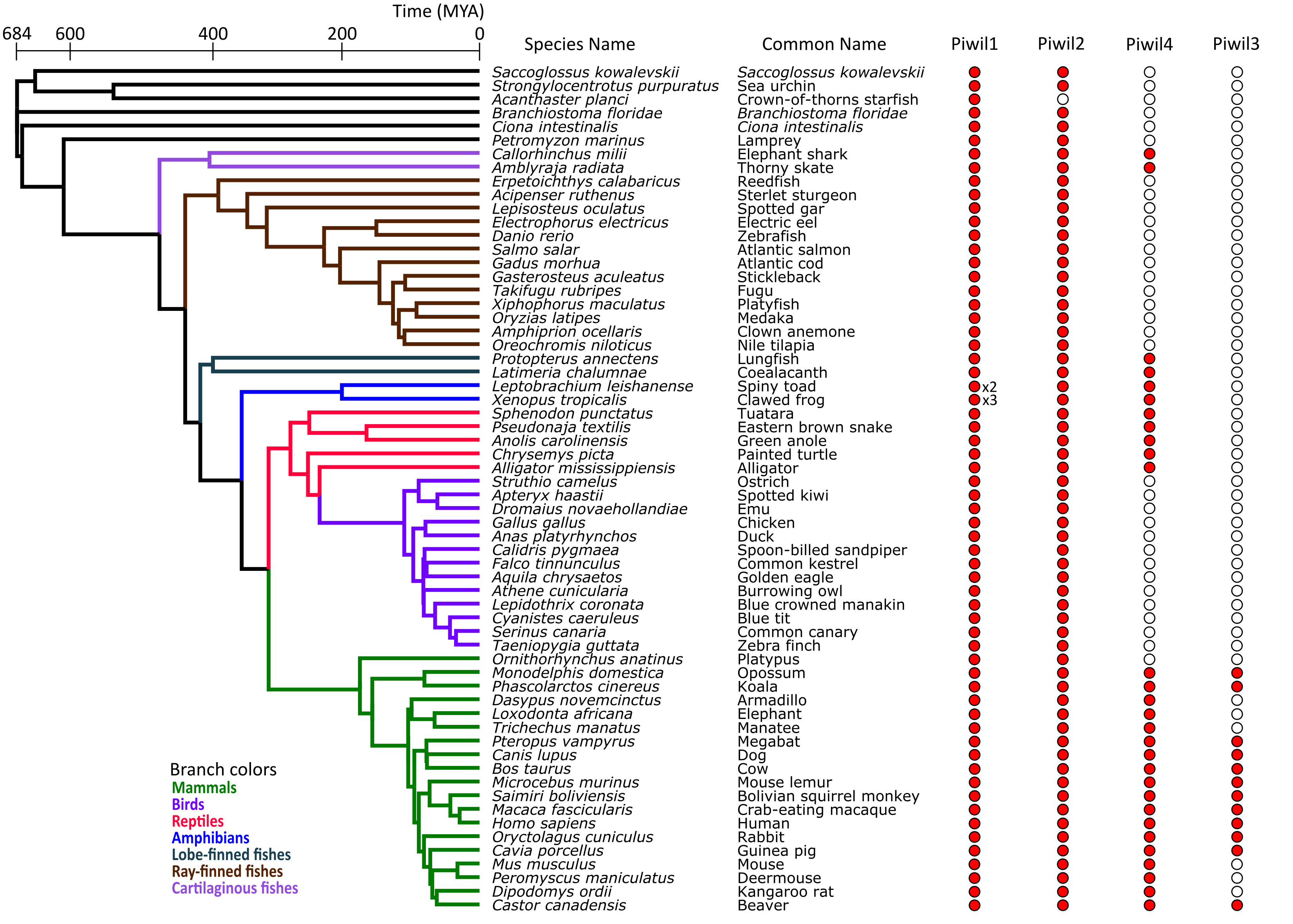

Supplement: Supplemental Information 1 — The phylogenetic tree was generated using TimeTree (Kumar et al. 2017) from a list of species used in the study. Branches are color coded by major gnathostome lineages. The presence of a Piwi paralog is indicated by a red circle and absent paralog is reflected by a white circle. An x2 or x3 by a Piwi indicates 2 or 3 copies of a paralog. Although Asterias rubens was used in the study, it is absent from the tree as As. rubens relationships are lacking from TimeTree, but the Piwi presence/absence mirrors Acanthaster planci and As. rubens is sister to Ac. planci (See Table S1). Kumar S, Stecher G, Li M, Knyaz C, Tamura K. 2018. MEGA X: molecular evolutionary genetics analysis across computing platforms. Molecular Biology and Evolution 35:15471549 DOI 10.1093/molbev/msy096. [file peerj-09-12451-s001.png]

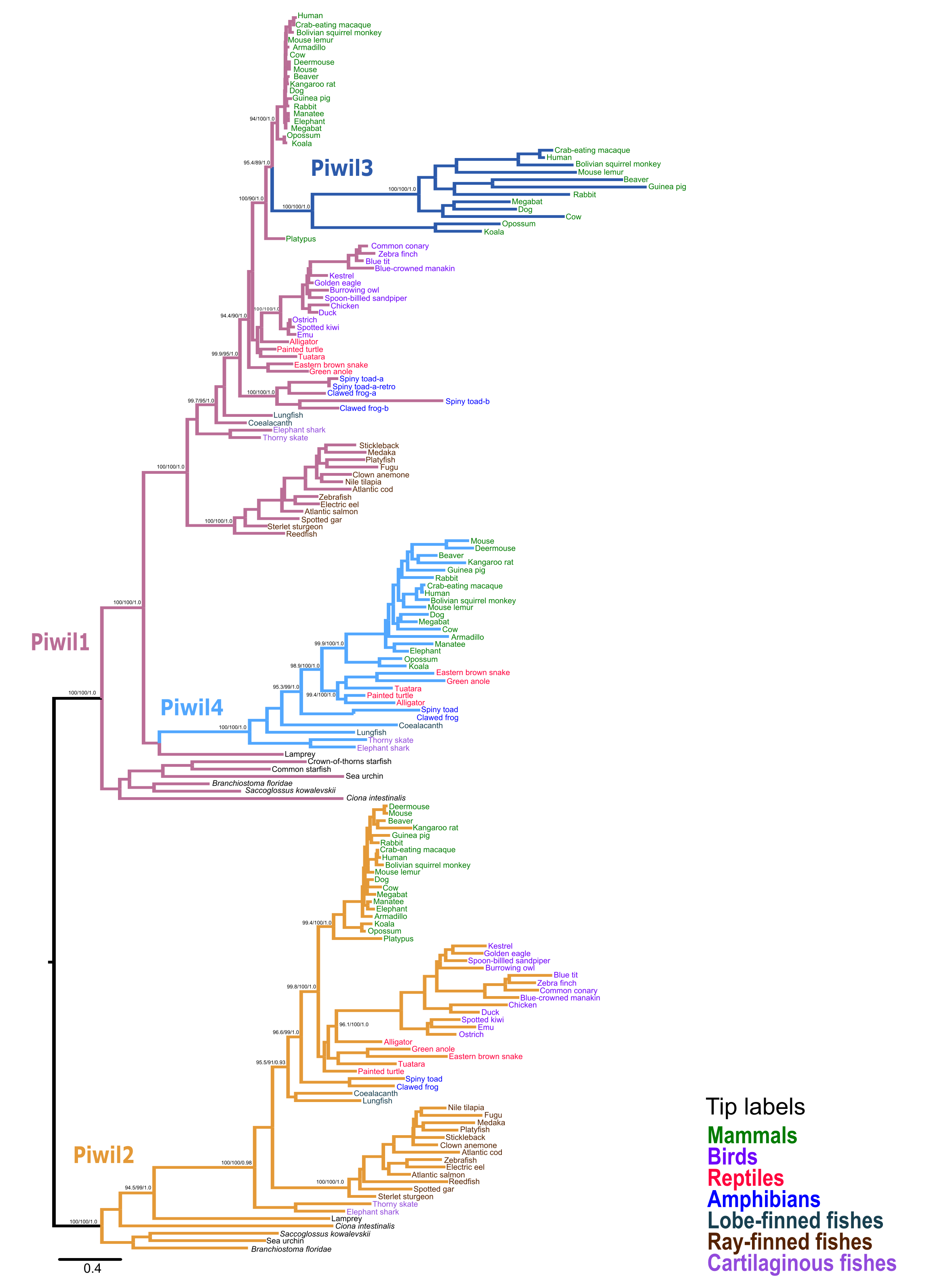

Supplement: Supplemental Information 2 — The IQ-Tree model chosen was JTT+F+R6 (JTT model using empirical base frequencies and a FreeRate model with 6 rate categories). Piwi paralogs are color coded on the tree and tip labels are color coded to by major gnathostome groups. The displayed tree was constructed with IQ-Tree2. Numbers next to nodes reflect nodal support derived from the ultrafast bootstrap routine/ SH-aLRT/and posterior probability from the Bayesian phylogeny that mirrored the displayed tree. [file peerj-09-12451-s002.png]

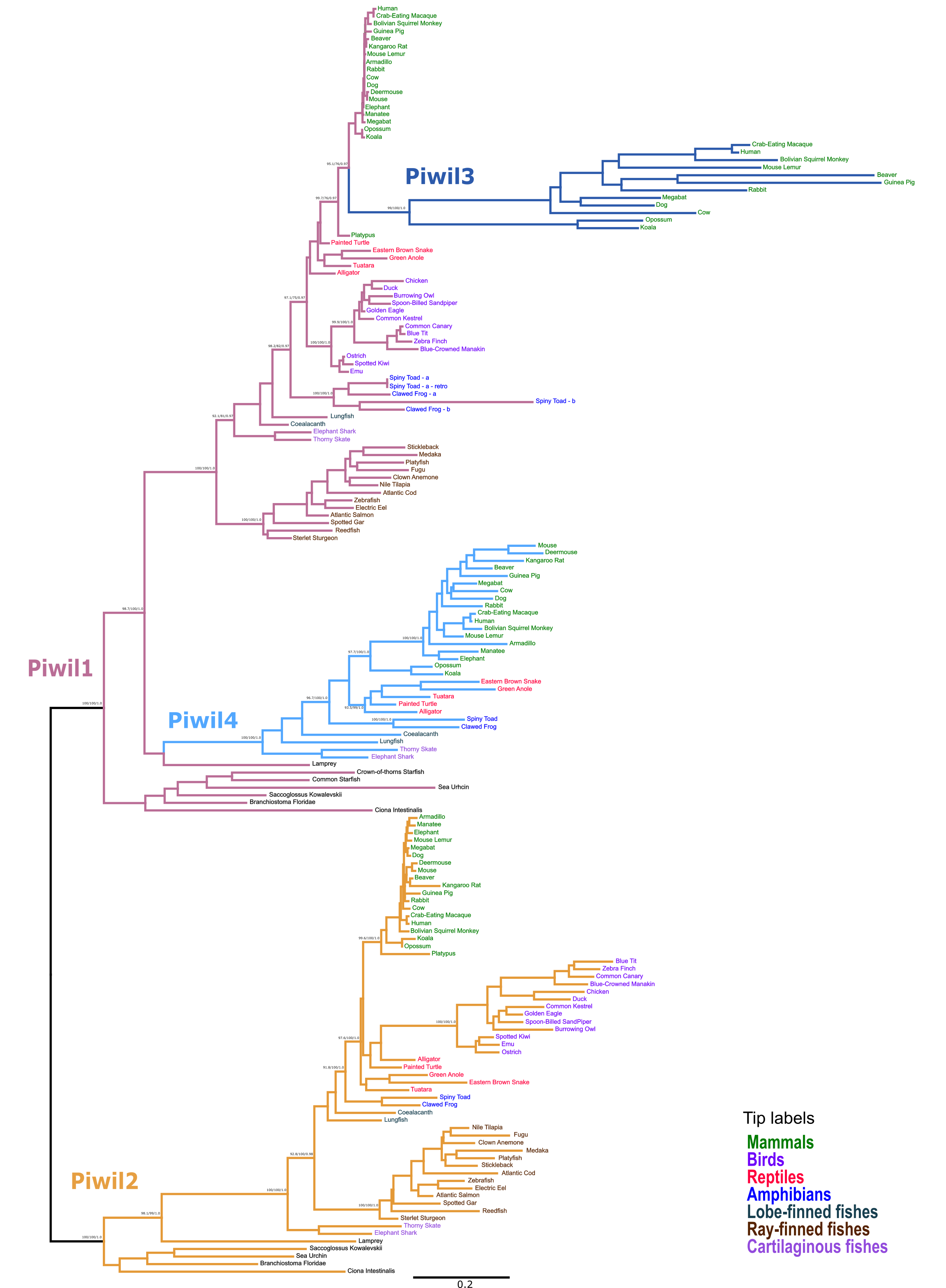

Supplement: Supplemental Information 3 — A phylogenetic tree reconstructed from the most alignable residues see Fig. S2 for additional description. The IQ-Tree model chosen was also JTT+F+R6. [file peerj-09-12451-s003.png]

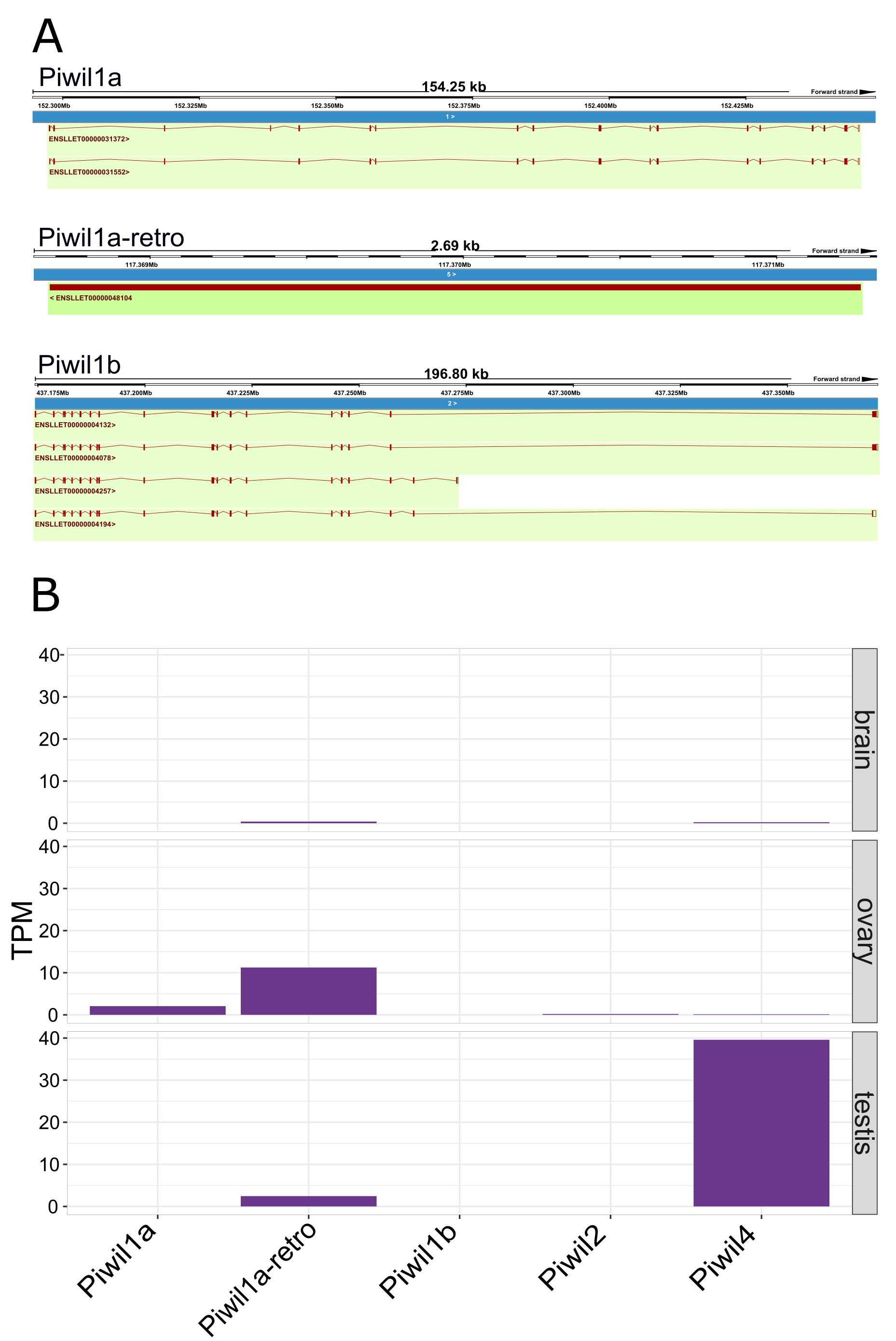

Supplement: Supplemental Information 4 — (A) Location and intron/exon boundaries of Piwil1 paralogs in the spiny toad. (B) Piwi expression among paralogs measured from available samples. [file peerj-09-12451-s004.png]

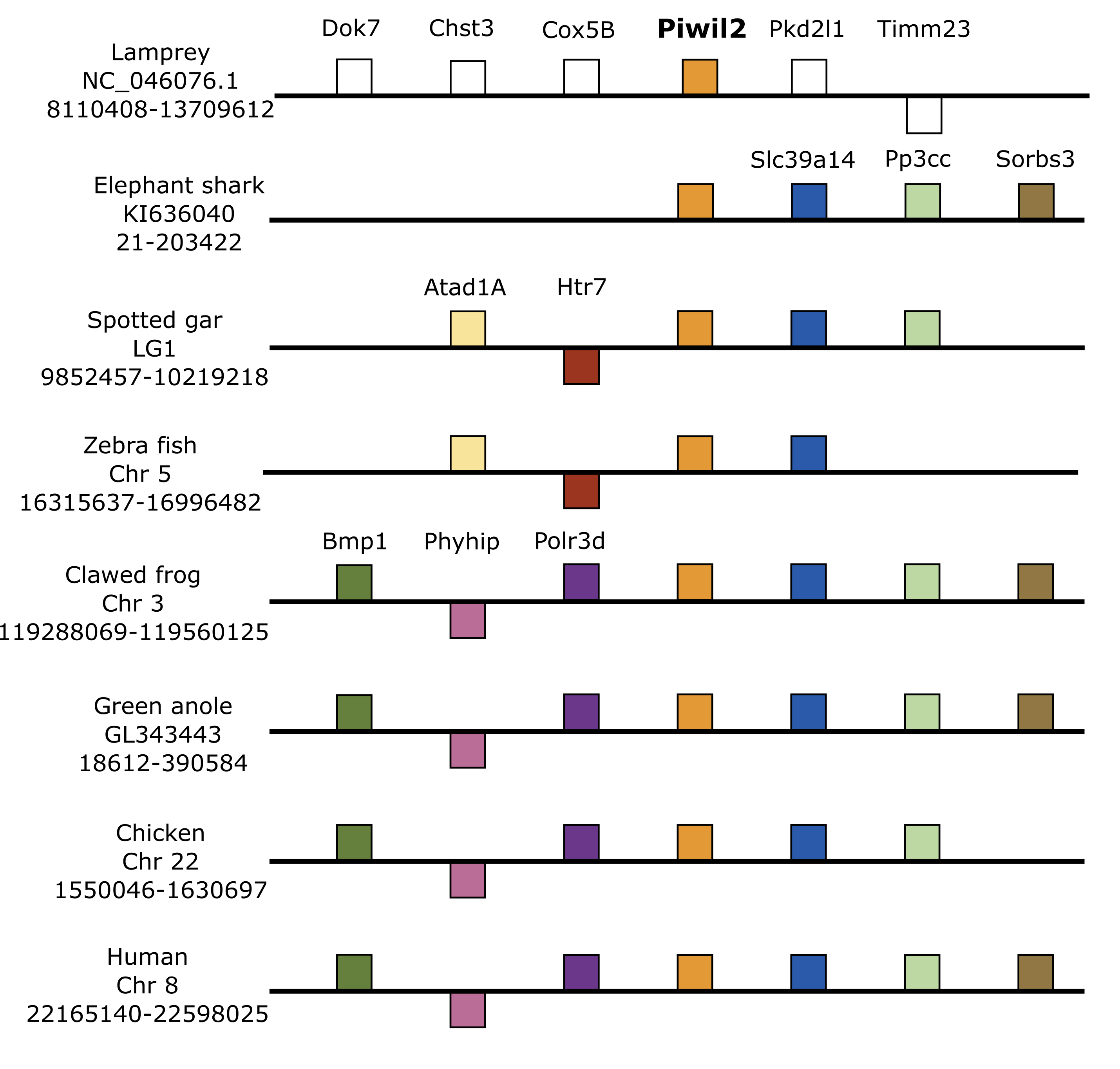

Supplement: Supplemental Information 5 — Organization of genes up and downstream of Piwil2. Distances are not drawn to scale. White boxes represent genes that are not homologous to any other genes in the synteny block. Boxes on top of the black line reflect genes in forward orientation relative to Piwi genes and boxes below the line are in the opposite orientation. [file peerj-09-12451-s005.png]

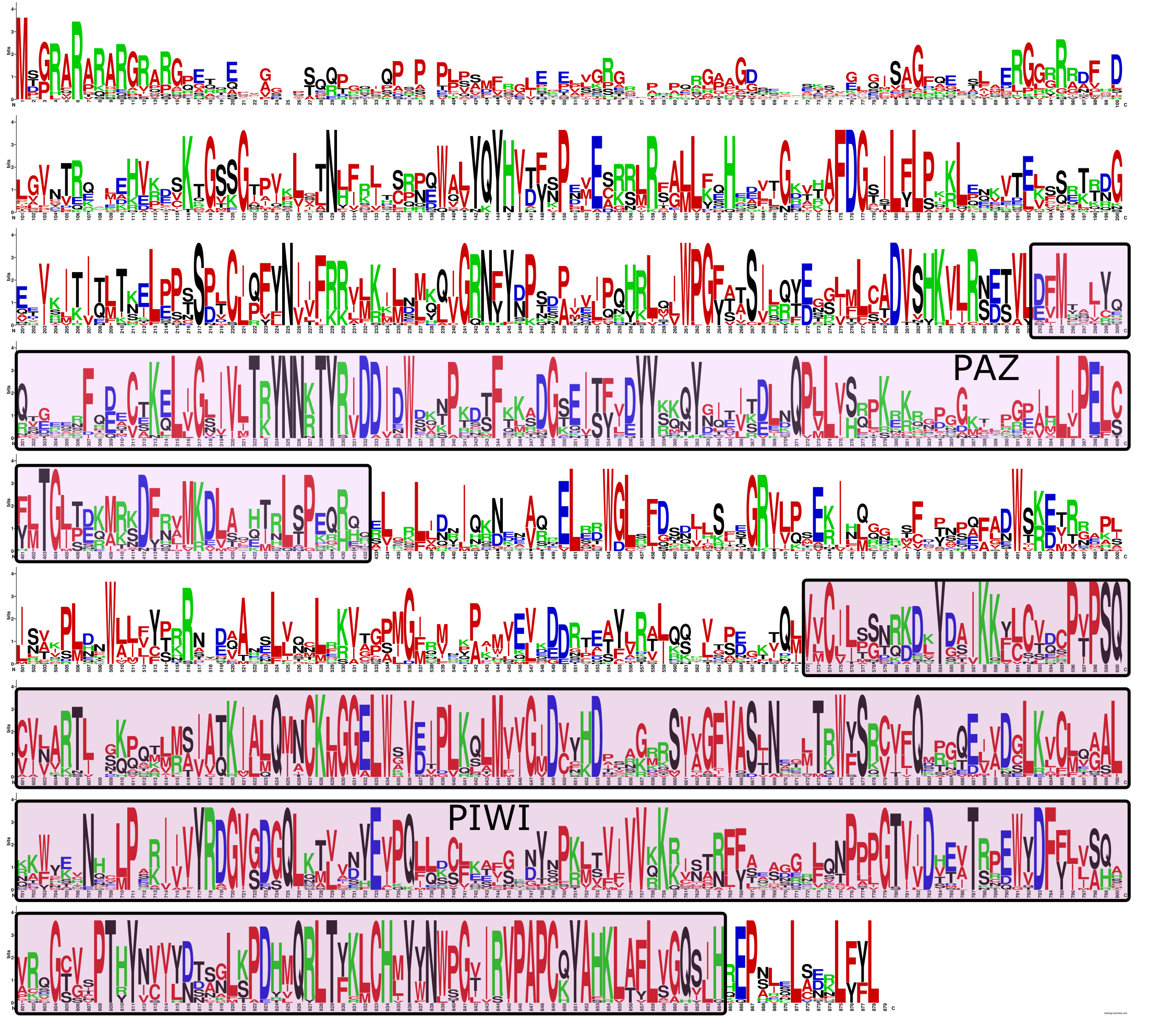

Supplement: Supplemental Information 6 — A sequence LOGO constructed from an amino acid alignment of all Piwi paralogs found in human, dog, chicken, spotted gar, and elephant shark. PAZ and PIWI domains are labeled. Highly gaped regions in the 5′section were removed prior to making the LOGO. [file peerj-09-12451-s006.png]

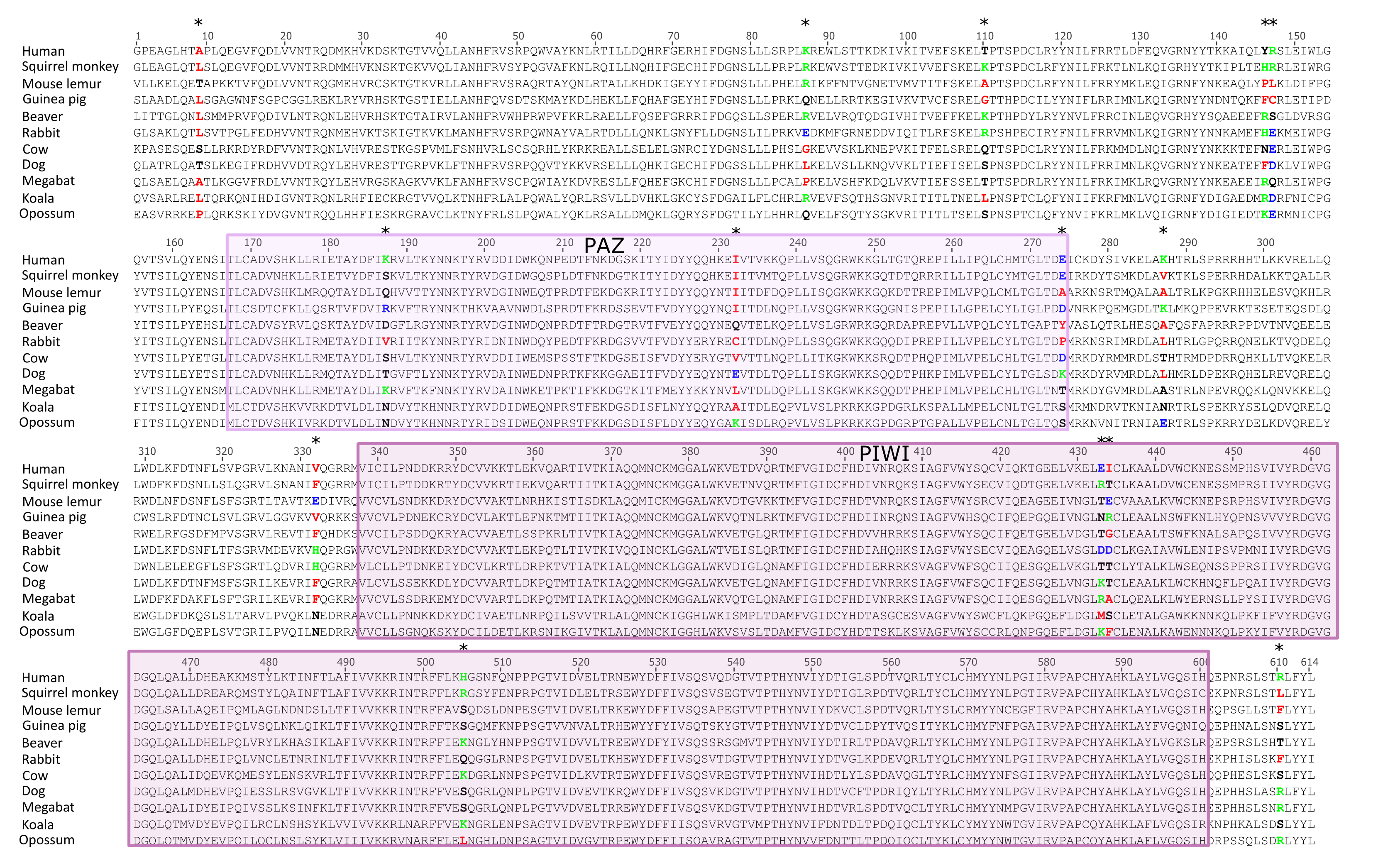

Supplement: Supplemental Information 7 — All sites tested for selection in codeml under model M2a presented in Fig. 3C. Sites labeled by an asterisk were found under selection with BEB >0.9. PAZ and PIWI domains are highlighted. [file peerj-09-12451-s007.png]

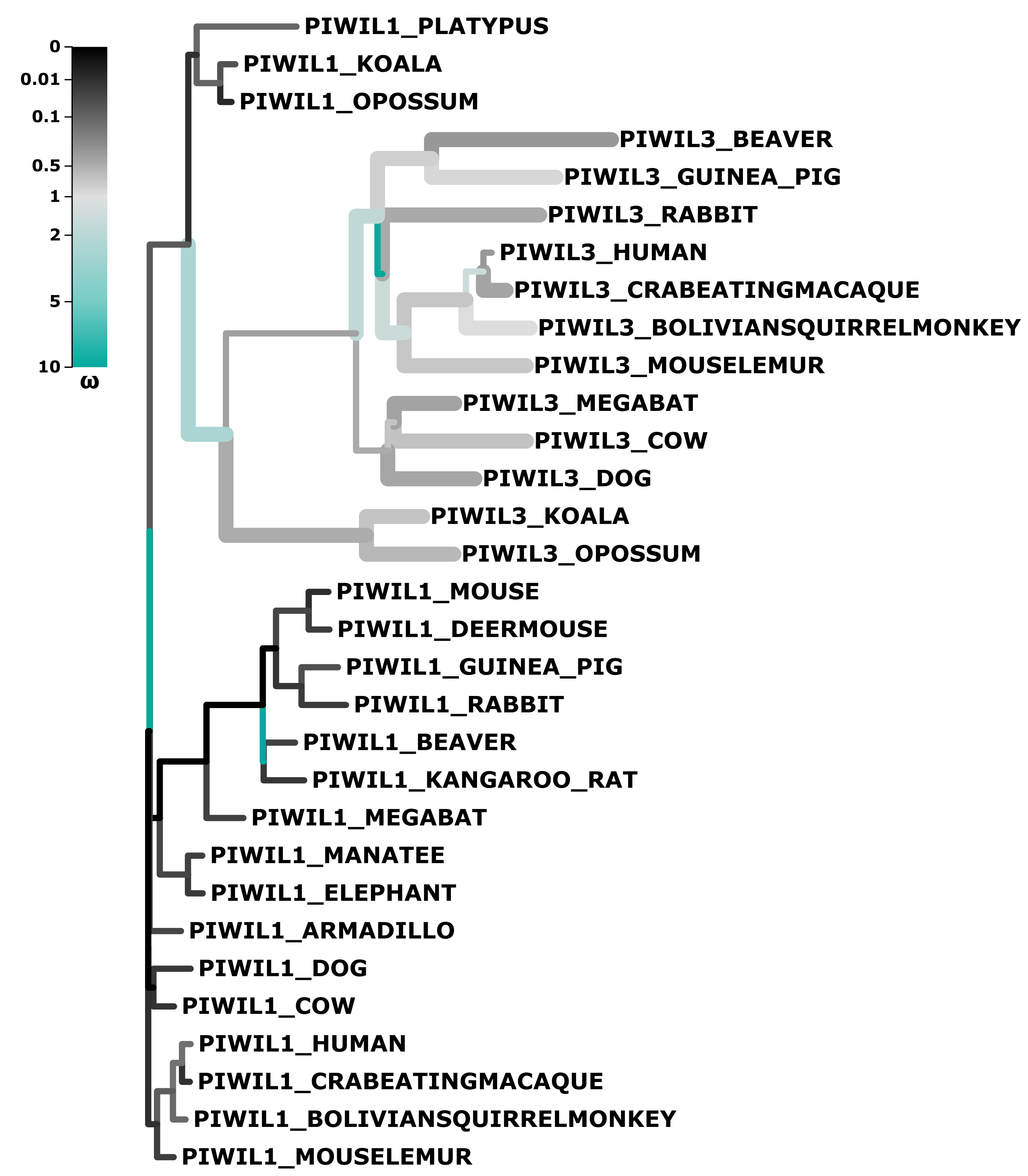

Supplement: Supplemental Information 8 — Piwil3 branches that had evidence of episodic diversifying selection are presented thicker. Significance was assessed using the LRT at a threshold of p ≤ 0.05, after correcting for multiple testing. [file peerj-09-12451-s008.png]
